# Autoimmune rheumatic diseases increase dementia risk in middle-aged patients: A nationwide cohort study

**DOI:** 10.1371/journal.pone.0186475

**Published:** 2018-01-05

**Authors:** Tzu-Min Lin, Wei-Sheng Chen, Jau-Jiuan Sheu, Yi-Hsuan Chen, Jin-Hua Chen, Chi-Ching Chang

**Affiliations:** 1 Division of Rheumatology, Immunology and Allergy, Department of Internal Medicine, Taipei Medical University Hospital, Taipei, Taiwan; 2 Division of Allergy, Immunology, and Rheumatology, Department of Internal Medicine, Taipei Veterans General Hospital, National Yang-Ming University, Taipei, Taiwan; 3 Department of Neurology, Taipei Medical University Hospital, Taipei, Taiwan; 4 Department of Neurology, School of Medicine, College of Medicine, Taipei Medical University, Taipei, Taiwan; 5 Biostatistics Center, College of Management, Taipei Medical University, Taipei, Taiwan; 6 Biostatistics Center and School of Health Care Administration, College of Management, Taipei Medical University, Taipei, Taiwan; 7 Division of Allergy, Immunology and Rheumatology, Department of Internal Medicine, School of Medicine, College of Medicine, Taipei Medical University, Taipei, Taiwan; Istituto di Ricovero e Cura a Carattere Scientifico Centro di Riferimento Oncologico della Basilicata, ITALY

## Abstract

**Objective:**

Dementia is a common neurological disease that substantially affects public health. A previous study revealed that dementia occurs when the body’s immune system attacks the cells of the brain, indicating that dementia may be similar to autoimmune rheumatic diseases (ARDs). In the current retrospective cohort study, we focused on middle-aged ARD patients (45 years or older) to investigate the association between ARDs in middle-aged people and dementia by using a nationwide population-based database in Taiwan.

**Method:**

Our study analyzed the medical data of the Taiwanese population from 2001 to 2012, with a follow-up period extending until the end of 2011. We identified middle-aged patients with ARDs by using the Taiwan National Health Insurance Research Database. We selected a comparison cohort from the general population that was randomly frequency-matched by age (in 5-year increments), sex, and index year and further analyzed the dementia risk by using a Cox regression model that considers sex, age, and comorbidities.

**Results:**

The study enrolled 34,660 middle-aged ARD patients (77% female, mean age = 59.8 years) and 138,640 controls. The risk of developing dementia was 1.18 times higher for middle-aged patients with ARDs compared with patients without ARDs after adjustment for age, sex, and comorbidities. Among the patients with ARDs, the subgroups with rheumatoid arthritis, systemic lupus erythematosus, and Sjögren syndrome (SS) were associated with a significantly higher dementia risk (adjusted hazard ratio [HR] 1.14, 95% confidence index [CI] 1.06–1.32; adjusted HR 1.07, 95% CI 0.86–1.34; adjusted HR 1.46, 95% CI 1.32–1.63, respectively). Furthermore, primary SS and secondary SS patients had the highest risks of dementia among all the ADR subgroups (adjusted HR 1.35, 95% CI 1.18–1.54; adjusted HR 1.67, 95% CI 1.43–1.95 respectively).

**Conclusion:**

This nationwide retrospective cohort study demonstrated that dementia risk is significantly higher in middle-aged patients with ARDs compared with the general population.

## Introduction

Dementia is a common disorder characterized by a decline in one or more cognitive functions that can impair the performance of daily activities [1]. Alzheimer disease (AD) is the most common type of dementia, accounting for 60% of all dementia cases. Other types of dementia are Parkinson disease dementia, frontotemporal dementia, and Lewy body dementia [2]. All types of neurodegenerative dementia are associated with neuroinflammation, which is characterized by reactive microgliosis, oxidative damage, and mitochondrial dysfunction. Autoimmune rheumatic diseases (ARDs), such as rheumatoid arthritis (RA), systemic lupus erythematosus (SLE), Sjögren syndrome (SS), progressive systemic sclerosis, polymyositis, dermatomyositis, vasculitis, and Behçet disease, also result from the dysregulation of the immune system and are characterized by progressive and systemic inflammation. A recent study suggested that dementia may occur when the body’s immune system attacks the cells of the brain, suggesting that some types of dementia may be similar to ARDs [3–4]. Moreover, multiple studies have revealed that ARDs increase the risk of vascular events such as ischemic stroke, acute myocardial infarction, and peripheral arterial occlusive disease [5–10]. Furthermore, several proinflammatory cytokines (IL-1b, IL-6, and TNF-α) participate in and increase the risk of dementia and AD as well as participate in the pathogenesis of ARDs [11–13]. However, most data on the association between ARDs and dementia are from studies with conflicting results that have used a case–control design or are small case series [14–19]. Therefore, the association between ARDs and dementia has not been fully established.

We hypothesize that ARDs predispose patients to the development of dementia. To verify this hypothesis, this cohort study examined the relationship between middle-aged patients (45 years or older) with ARDs and dementia by analyzing a large population-based database.

## Methods

### Data sources

The National Health Insurance (NHI) program was initiated in 1995 to provide thorough healthcare for citizens and residents of Taiwan. Enrollment in this program is mandatory, resulting in a coverage rate of almost 99% [20]. The Taiwan National Health Insurance Research Database (NHIRD), which is maintained by the Department of Health and the National Health Research Institutes of Taiwan, comprises comprehensive medical care information available for research purposes. This database provides basic information about each person insured by the NHI, including patient characteristics, records of outpatient visits, hospital admissions, drug prescriptions, and disease status and management. The diagnostic codes used are formatted in accordance with the International Classification of Diseases, Ninth Revision, Clinical Modification (ICD-9-CM). At the time of this study, the NHIRD was electronic with patients’ personal information being encrypted for privacy protection. The study was approved by the Institutional Review Board of Taipei Medical University (approval number N201509007) and was performed according to the relevant guidelines. Informed consent of the study patients was not required because the dataset used in this study comprised deidentified secondary data released for research purposes. Patient consent was not required to access the NHIRD.

### Study patients

Patients diagnosed with ARDs between 2001 and 2012 were identified from the catastrophic illness registry in the NHIRD. In Taiwan, patients with ARDs are eligible for a catastrophic illness certificate after a rheumatology specialist diagnoses them on the basis of clinical manifestations, laboratory data, and the criteria set by the American College of Rheumatology, which is reviewed by rheumatologists commissioned by the NHI. Thus, the catastrophic illness patient data are highly accurate and reliable. Several autoimmune diseases are defined as catastrophic illnesses by the NHI with the related certification requiring precise fulfillment of the following classification criteria: the American College of Rheumatology (ACR) 1997 revised criteria for SLE (ICD-9-CM: 710.0) [21]; the American Rheumatism Association 1987 revised criteria for RA (ICD-9-CM: 714.0) [22]; the ACR criteria for systemic sclerosis (ICD-9-CM: 710.1) [23]; the American–European Consensus Group 2002 revised criteria for SS (ICD-9-CM: 710.2) [24]; the Bohan and Peter 1975 criteria for polymyositis and dermatomyositis (ICD-9-CM: 710.3) [25,26]; the International Study Group 1990 criteria for Behçet disease (ICD-9-CM: 136.1) [27]; and the ACR 1990 criteria for temporal arteritis (ICD-9-CM: 443.1) [28], granulomatosis polyangiitis (ICD-9-CM: 446.4) [29], and Takayasu arteritis (ICD-9-CM: 446.7) [30]. The date of the earliest ARD diagnosis was used as the index date. Patients with a history of dementia or who were younger than 45 years were excluded. Finally, 34,660 patients with ARDs were selected as the study patients and were designated as the ARD cohort. For each ARD patient, four non-ARD patients were randomly selected from the same study period according to the same exclusion criteria and were frequency-matched with the ARD patients according to age and sex to construct the non-ARD cohort, which comprised 138,640 patients.

### Outcome measurement and comorbidities

Each study patient was followed until receiving their first diagnosis of any type of dementia by a neurologist during two visits to the outpatient department or one hospital admission. The dementia types included Alzheimer disease (ICD-9-CM: 331.0), arteriosclerotic dementia (ICD-9-CM: 290.4), and unspecified dementia (ICD-9-CM: 290.0–290.3, 294.1, 331.1–331.2, and 331.82). The patients who recorded ICD-9-CM 290.4 in the claims data were classified as having vascular dementia. All remaining patients with dementia who did not belong to the vascular dementia group were defined as having nonvascular dementia. We also differentiated patients with Alzheimer’s disease from those with non-vascular dementia by searching for ICD9-CM code 331.0. The patient was considered lost to follow-up on death, withdrawal from the database, or the end of 2012. At the baseline, major comorbidities such as diabetes (ICD-9-CM 250), hyperlipidemia (ICD-9-CM 272), hypertension (ICD-9-CM 401–405), heart failure (ICD-9-CM 428, I50.2, I50.3), cardiovascular disease (ICD-9 codes 393–398, 410–414, 420–429, 440–449, 451–459), stroke (ICD-9-CM 430–438), major psychosis or a substance-related disorder (ICD-9-CM codes 291–299, 303–305), and traumatic brain injury (ICD-9 CM codes 801–804, 850–854 were considered covariates.

### Statistical analyses

This was a nationwide retrospective cohort study. The baseline characteristics were sex, age, and certain comorbidities. The baseline characteristics were matched between the ARD and non-ARD patients according to age and sex and were compared using a chi-square test for categorical variables and a t-test for continuous variables. We observed the time-to-event data in the ARD and non-ARD cases and used a multiple Cox proportional hazards model to explore the association between dementia and the ARDs adjusted for sex, age, and comorbidities. The adjusted hazard ratios (HRs) indicated that after adjusting for covariates, the ARD cases had a higher dementia risk than did the non-ARD cases when HR > 1. The 95% confidence intervals (CIs) of the HRs were also calculated.

To perform a stratified analysis, we separately calculated the incident rate ratio and HRs adjusted for sex, age, comorbidities, and some types of ARDs (including RA, SLE, and SS) for the age groups of less than 65 years and 65 or more years. We used the Kaplan–Meier method to calculate the incidence rates for the ARD, RA, primary SS, secondary SS, and SLE groups over the follow-up period. We then plotted the results in cumulative incidence plots with each comparison group’s cumulative incidence rate. All analyses were performed using SAS version 9.4 (SAS Institute, Cary, NC).

## Results

A total of 34,660 cases of ARDs and 138,640 matched control cases were selected from the NHIRD during the defined period of interest. Of the ARD and the non-ARD patients, 39% were 45–54 years of age and 77% were female (Table 1). The ARD cohort was more likely than the non-ARD cohort to experience diabetes (14.54% vs. 12.98%, p < 0.001), hyperlipidemia (16.66% vs. 14.21%, p < 0.001), psychosis (4.09% vs. 2.4%, p < 0.001), heart failure (4.71% vs. 1.91%, p < 0.001), hypertension (34.99% vs. 27.69%, p < 0.001), stroke (7.18% vs. 4.85%, p < 0.001), traumatic brain injury (1.47% vs. 1.23%, p < 0.001), or cardiovascular disease (34.01% vs. 16.42%, p < 0.001). The mean follow-up period was 5.97 years (standard deviation [SD] 3.05) and 6.37 years (SD 2.95) for the ARD and the non-ARD cohorts, respectively.

**Table 1:** Baseline characteristics of the ARD patient group and age- and sex-matched comparison group.

Group	Comparison group		ARD patient group		
	(N = 138,640)		(N = 34,660)		
Variable	n	(%)	N	(%)	p value*
Sex					1.000
Male	31,448	(22.68)	7862	(22.68)	
Female	107,192	(77.32)	26,798	(77.32)	
Age, mean (SD)	59.80	(10.16)	59.80	(10.11)	0.988
Age group					0.996
45–54	54,924	(39.62)	13,756	(39.69)	
55–64	41,798	(30.15)	10,432	(30.10)	
65–74	28,838	(20.80)	7203	(20.78)	
≥75	13,080	(9.43)	3269	(9.43)	
Comorbidities					
Diabetes	17,989	(12.98)	5039	(14.54)	<0.001
Hyperlipidemia	19,694	(14.21)	5575	(16.66)	<0.001
Psychosis	3326	(2.40)	1419	(4.09)	<0.001
Heart failure	2652	(1.91)	1634	(4.71)	<0.001
Hypertension	38,384	(27.69)	12,128	(34.99)	<0.001
Stroke	6729	(4.85)	2488	(7.18)	<0.001
Traumatic brain injury	1712	(1.23)	511	(1.47)	<0.001
Cardiovascular disease	22,761	(16.42)	11,787	(34.01)	<0.001

* p values were calculated using a chi-square test for categorical variables and a t-test for continuous variables

Table 2 lists the dementia incidence densities for the ARD and non-ARD cohorts. During the observation period, 4280 patients in the non-ARD cohort (incidence rate of 48.43 per 10,000 person-years) and 1305 patients in the ARD cohort (incidence rate of 63.08 per 10,000 person-years) developed dementia. When stratified by sex, age, and comorbidities, the patients in the ARD cohort, particularly male patients (HR 1.23, 95% CI 1.09–1.4) and patients aged ≥75 years (HR 1.26, 95% CI 1.13–1.40), were associated with an increased dementia risk.

**Table 2:** Incidence of dementia and Cox model results for the ARD patient group and the comparison group.

	Comparison group		ARD patient group			
Characteristics	Event (%)	Incidence§	Event (%)	Incidence§	IRR	Adjusted HR^a^ (95% CI)
All	4280(3.09)	48.43	1305 (3.77)	63.08	1.30	1.18 (1.11–1.26)***
Sex						
Female	3211 (3.00)	46.75	969 (3.62)	59.58	1.27	1.18 (1.10–1.27)***
Male	1069 (3.40)	54.30	336 (4.27)	75.94	1.40	1.23 (1.09–1.40)**
Age						
<65	828 (0.86)	12.98	259 (1.07)	16.81	1.30	1.06 (0.92–1.22)
65–74	1894 (6.57)	107.23	567 (7.87)	145.00	1.35	1.20 (1.09–1.32)***
≥75	1558 (11.91)	225.59	479 (14.65)	349.00	1.55	1.26 (1.13–1.40)***
Diabetes						
No	3164 (2.62)	40.53	985 (3.33)	54.82	1.35	1.21 (1.12–1.30)***
Yes	1116 (6.20)	108.15	320 (6.35)	117.57	1.09	1.06 (0.94–1.21)
Hyperlipidemia						
No	3408 (2.87)	44.33	1061 (3.67)	60.96	1.38	1.23 (1.14–1.32)***
Yes	872 (4.43)	75.87	244 (4.23)	74.28	0.98	1.00 (0.87–1.16)
Psychosis						
No	3962 (2.93)	45.79	1164 (3.50)	58.40	1.28	1.20 (1.12–1.28)***
Yes	318 (9.56)	171.32	141 (9.94)	186.48	1.09	1.06 (0.87–1.30)
Hypertension						
No	1912 (1.91)	29.10	568 (2.52)	40.55	1.39	1.32 (1.20–1.45)***
Yes	2368 (6.17)	104.50	737 (6.08)	110.31	1.06	1.05 (0.97–1.14)
Stroke						
No	3437 (2.61)	40.55	1050 (3.26)	53.95	1.33	1.26 (1.18–1.35)***
Yes	843 (12.53)	232.47	255 (10.25)	207.99	0.89	0.90 (0.78–1.04)
Traumatic brain injury						
No	4158 (3.04)	47.61	1247 (3.65)	61.07	1.28	1.17 (1.10–1.25)***
Yes	122 (7.13)	117.10	58 (11.35)	214.73	1.83	1.60 (1.16–2.21)**
Cardiovascular disease						
No	2731 (2.36)	36.49	649 (2.84)	46.08	1.26	1.29 (1.18–1.40)***
Yes	1549 (6.81)	114.43	656 (5.57)	99.33	0.87	1.03 (0.94–1.13)

§ Incidence per 10,000 person-years

*: p value for HR < 0.05

**: p value for HR < 0.01

***: p value for HR < 0.001

a HR adjusted by age group, sex, and comorbidities

We further divided the ARD cohort into RA, SLE, primary SS, secondary SS, and other ARDs subgroups. An HR of 1.23 (95% CI 1.15–1.31, p < 0.001) was obtained for the ARD cohort (Table 3), and the adjusted HRs for dementia for the RA, SLE, and SS subgroups were 1.14, 1.07, and 1.46, respectively. Furthermore, primary SS and secondary SS patients had the highest dementia risk among all patients with ARDs, with the adjusted HRs for dementia being 1.35 and 1.67, respectively. Specifically, the secondary SS subgroup had the highest dementia risk overall (HR 1.67, 95% CI 1.43–1.95, p < 0.001). In the age group of less than 65 years, the adjusted HRs for dementia for the RA, SLE, primary SS, secondary SS, and other ARDs subgroups were 1.08, 0.88, 1.49, 1.64, and 0.95, respectively. In this age group, secondary SS had the highest risk of dementia (HR 1.64, 95% CI 1.2–2.25, p < 0.01). In the age group of 65 years or older, the adjusted HRs for dementia for the RA, SLE, primary SS, secondary SS, and other ARDs subgroups were 1.15, 1.19, 1.35, 1.66, and 1.33, respectively. In this age group also, secondary SS had the highest risk of dementia (HR 1.66, 95% CI 1.39–1.99, p < 0.001).

**Table 3:** Incidence of dementia and Cox model results for the ARD patient group and comparison group.

Characteristics	N	Event (%)		Incidence§	Adjusted HR^a^ (95%CI)
**All**					
Comparison group	138640	4280	(3.09)	48.43	1.00
All ARD patients	34660	1305	(3.77)	63.08	1.23 (1.15–1.31)***
RA	19556	713	(3.65)	58.07	1.14 (1.06–1.32)**
SLE	3062	82	(2.68)	46.32	1.07 (0.86–1.34)
SS	8449	408	(4.83)	87.11	1.46 (1.32–1.63)***
Primary SS	4756	238	(5.00)	95.96	1.35 (1.18–1.54)***
Secondary SS	3693	170	(4.60)	77.14	1.67 (1.43–1.95)***
Other ARDs	3593	102	(2.84)	52.11	1.21 (0.99–1.48)
**Age < 65**					
Comparison group	96722	828	(0.86)	12.98	1.00
All ARD patients	24188	259	(1.07)	16.81	1.16 (1.00–1.33)*
RA	13417	142	(0.79)	15.78	1.08 (0.90–1.29)
SLE	2299	17	(0.74)	11.70	0.88 (0.54–1.42)
SS	5802	79	(1.36)	23.47	1.57(1.24–1.98) ***
Primary SS	3057	37	(1.21)	22.39	1.49(1.07–2.07)*
Secondary SS	2745	42	(1.53)	24.51	1.64 (1.20–2.25)**
Other ARDs	2670	21	(0.79)	13.23	0.95 (0.61–1.47)
**Age ≥ 65**					
Comparison group	41918	3452	(8.24)	140.50	1.00
All ARD patients	10472	1046	(9.99)	198.00	1.25 (1.16–1.34)***
RA	6139	571	(9.30)	174.17	1.15 (1.06–1.26)**
SLE	763	65	(8.52)	205.06	1.19 (0.93–1.53)
SS	2647	329	(12.43)	249.74	1.45(1.30–1.63) ***
Primary SS	1699	201	(11.83)	242.92	1.35 (1.17–1.55)***
Secondary SS	948	128	(13.50)	261.27	1.66 (1.39–1.99)***
Other ARDs	923	81	(8.52)	218.88	1.33 (1.06–1.66)*

§ Incidence per 10,000 person-years

*: p value for HR < 0.05

**: p value for HR < 0.01

***: p value for HR < 0.001

a HR adjusted by age group, sex, and comorbidities

**Fig 1:**
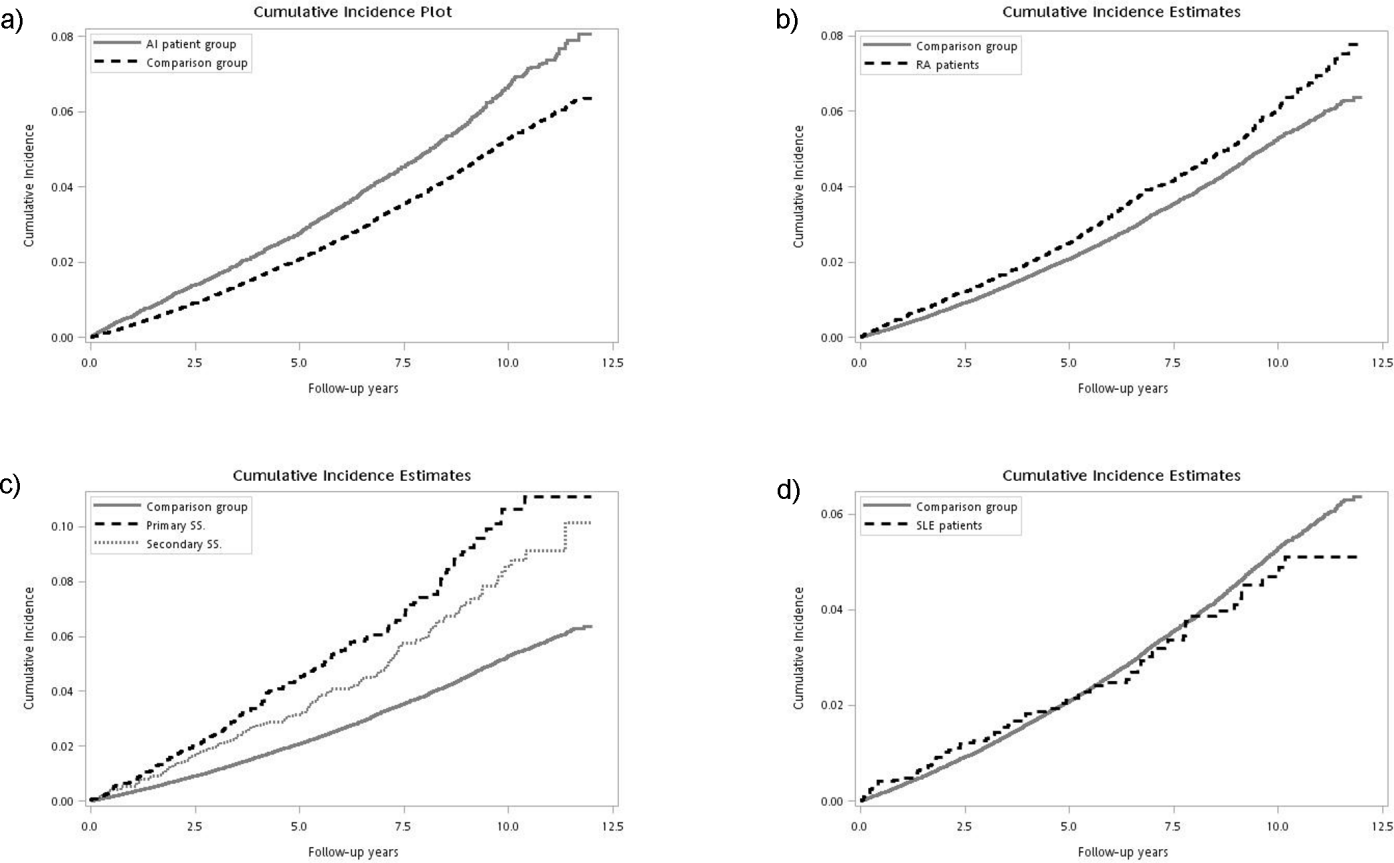
Cumulative incidence plot of dementia among ARD and non-ARD patient group. (a) presents a comparison of the cumulative incidence of dementia for the ARD and non-ARD patient groups. (b) presents a comparison of the cumulative incidence of dementia for RA and non-ARD cohorts. (c) presents a comparison of the cumulative incidence of dementia for SS (primary SS and secondary SS) and non-ARD cohorts. (d) presents a comparison of the cumulative incidence of dementia for SLE and non-ARD cohorts.

Fig 1(A) presents a comparison of the cumulative incidence of dementia for the ARD and non-ARD patient groups. The incidence of dementia (log rank test, p < 0.001) was significantly higher for patients in the ARD cohort than it was for patients without ARDs. Fig 1(B)–1(D) presents a comparison of the cumulative incidence of dementia for the subgroups of the ARD and non-ARD cohorts. Except for the SLE subgroup, the incidence of dementia (log rank test, p < 0.001) was significantly higher for the patients in the subgroups of the ARD cohort than it was for the non-ARD cohort.

## Discussion

To the best of our knowledge, this is the first nationwide population-based study evaluating the relationship between middle-aged patients with ARDs and dementia. In this study, the overall incidence rate of dementia was 30% higher for the ARD cohort than for the non-ARD cohort, with an HR of 1.18 after adjustment for age, sex, and comorbidities. Moreover, ARD subgroups such as the RA and SS subgroups were associated with a significantly higher risk of dementia than was the non-ARD cohort. We therefore postulate that patients with ARDs (except SLE and vasculitis) have an increased risk of dementia.

Young-onset dementia (YOD) is defined as a neurological syndrome that affects the behavior and cognition of patients aged 45–64 years. In the present study, the patients were divided into two age groups, <65 and ≥65 years, for analysis. The age of the patients and follow-up suggested that the association is not restricted to YOD and also involves late-onset dementia. Speculations of a potential association between ARDs and neuropsychiatric affections, including affective disorder, neurotic disorder, personality disorder, dementia, and delirium, have increased [31,32]. The present study highlighted major psychosis or substance-related disorders (ICD-9-CM: 291–299 and 303–305) as covariates to differentiate between psychosis and dementia in SLE. Several substantial interactions between covariate conditions and ARDs have been reported to increase the risk of dementia. Microgial cell activation is a major component of neuroinflammation in degenerative dementia [33–35]. An activated microgial cell can be divided as either M1 (classical phenotype) or M2 (alternatively activated phenotype) [36]. Microglia develop into an M1 phenotype in the presence of interferon and tumor necrosis factor and release massive inflammatory cytokines such as IL1β, IL-12, TNF-α, and inducible nitric oxide synthase. These inflammatory cytokines have also been observed in ARDs. Additionally, the M2 phenotype develops in the presence of IL-4 and IL-13 and has an anti-inflammatory profile. The switch between the M1 and M2 phenotype is a dynamic process and is dependent on the presence of a peripheral inflammation state [37]. However, a specific type of M1 is predominant in ARDs, as reported by Jimenz et al. [35,38], who identified a distinctive shift from M2 to M1 in brains with AD. Therefore, glial cells can be activated by systemic inflammation and consequently deteriorate, presenting clinical symptoms of AD and Parkinson disease [39]. Neuroinflammation results in synaptic impairment, and neuronal death and contributes to neurodegeneration within the brain [40]. Therefore, middle-aged patients with ARDs may have an increased risk of dementia. Some animal models of AD [41,42] and histochemical analyses of human brain serial sections [43] also indicate an aggregation of activated microglia around amyloid plaques in animal [41] and human brains [44–47], respectively. Soluble Aβ may be involved [47] and may trigger neuroinflammation at the BBB level [48], indicating that inflammation is an early process in AD pathogeny.

Kang et al. [49] demonstrated that after adjustment for demographics and comorbidities, the dementia risk did not differ significantly in their ARD and comparison groups. Furthermore, neuroinflammation may have some protective effects against ARD. Additionally, de Simone et al. investigated the phospholipid phosphatidylserine expressed on the surface of apoptotic neurons and reported that its presence may induce a shift in the microglial expression of cytokines from being deleteriously inflammatory (IL-1, TNF-α, NO) to protective (TGF-β and NGF) [50]. Toll-like receptors (TLRs) are believed to promote the proinflammatory pathways. However, studies have reported that TLR2 and TLR4 are present in amyloid plaques [51] and are involved in the uptake of Aβ and other aggregated proteins, promoting their clearance from the central nervous system [52]. Another toll-like receptor, TLR3, can enhance neuronal survival and endothelial cell growth, promoting neuroprotective responses [53]. These findings may be due to neuroinflammation having both neurodestructive and neuroprotective effects. The hypothesis that inflammation leads to dementia implies a predominance of the neurodestructive effects over the neuroprotective effects [54].

Among the ARD subgroups in this study (RA, SLE, primary SS, secondary SS, and other ARDs), the secondary SS subgroup had the highest dementia risk after adjustment for demographics and comorbidities. Conversely, the SLE subgroup had the lowest dementia risk compared with the other ARD subgroups. These findings are consistent with those of Trysberg et al. [47–49], who reported low levels of amyloid β in SLE patients. This may be a consequence of diminished production of the amyloid precursor protein, which is believed to be mediated by heavy anti-inflammatory or immunosuppressive therapy. Therefore, the effects of anti-inflammatory or immunosuppressive therapy on patients deserves consideration.

A strength of our study was the use of a nationwide population-based database with sufficient sample size and statistical power. However, our study had several limitations. First, although the NHIRD includes data on patients that received treatment for ARDs and dementia, in Taiwan, most ARD patients with a catastrophic illness certificate have been treated with anti-inflammatory or immunosuppressive therapy. Additionally, in Taiwan, stopping anti-inflammatory or immunosuppressive therapy in ARD patients with a catastrophic illness certificate is unacceptable. Therefore, we could not adjust for treatment-related effects in our study. Although some studies have revealed that NSAID use reduces the risk of dementia in patients with RA, our data demonstrated an increased risk of dementia (adjusted HR: 1.14) in the RA subgroup. Such conflicting results may have been obtained because the present study did not consider the effects of NSAID use. Second, the NHIRD does not contain parameters such as clinical severity, laboratory data, body mass index, smoking habits, intelligence, and education level, all of which have been also associated with ARDs and dementia [9–10]. Third, dementia is more likely to occur in the elderly; however, patients of varying ages were enrolled to study the different ARDs. Therefore, we did not fully account for the effects of age in our study. Future analysis of each type of ARD may yield more concise results.

In conclusion, this nationwide population-based retrospective cohort study determined that middle-aged patients with ARDs (excluding SLE and vasculitis) have an increased risk of dementia. Among the ARD subgroups, the RA and SS subgroups were associated with a significantly higher risk of dementia, with both the primary and secondary SS subgroups having the highest overall risk of dementia. The underlying mechanism of these results is not fully understood and warrants further research.
